# Mind the “Vaccine Fatigue”

**DOI:** 10.3389/fimmu.2022.839433

**Published:** 2022-03-10

**Authors:** Zhaohui Su, Ali Cheshmehzangi, Dean McDonnell, Claudimar Pereira da Veiga, Yu-Tao Xiang

**Affiliations:** ^1^School of Public Health, Institute for Human Rights, Southeast University, Nanjing, China; ^2^Department of Architecture and Built Environment, Architecture and Urban Design, Faculty of Science and Engineering, University of Nottingham Ningbo China, Ningbo, China; ^3^Network for Education and Research on Peace and Sustainability, Hiroshima University, Hiroshima, Japan; ^4^Department of Humanities, South East Technological University, Carlow, Ireland; ^5^School of Management—PPGOLD, Federal University of Parana—UFPR, Curitiba, Brazil; ^6^Unit of Psychiatry, Department of Public Health and Medicinal Administration, University of Macau, Macao, Macao SAR, China; ^7^Institute of Translational Medicine, Faculty of Health Sciences, University of Macau, Macao, Macao SAR, China; ^8^Centre for Cognitive and Brain Sciences, University of Macau, Macao, Macao SAR, China; ^9^Institute of Advanced Studies in Humanities and Social Sciences, University of Macau, Macao, Macao SAR, China

**Keywords:** COVID-19, vaccination, vaccine fatigue, public health, vaccine communications

## Abstract

**Background:**

Omicron scares and speculations are gaining momentum. Amid the nonstop debates and discussions about COVID-19 vaccines, the “vaccine fatigue” phenomenon may become more prevalent. However, to date, no research has systematically examined factors that shape people’s vaccine fatigue. To bridge the research gap, this study aims to investigate the antecedents that cause or catalyze people’s vaccine fatigue.

**Methods:**

A narrative literature review was conducted in PubMed, Scopus, and PsycINFO to identify factors that shape people’s vaccine fatigue. The search was completed on December 6, 2021, with a focus on scholarly literature published in English.

**Results:**

A total of 37 articles were reviewed and analyzed. Vaccine fatigue was most frequently discussed in the context of infectious diseases in general at the pre-vaccination stage. Vaccine fatigue has been identified in the general public, the parents, and the doctors. Overall, a wide range of antecedents to vaccine fatigue has been identified, ranging from the frequency of immunization demands, vaccine side effects, misconceptions about the severity of the diseases and the need for vaccination, to lack of trust in the government and the media.

**Conclusion:**

Vaccine fatigue is people’s inertia or inaction towards vaccine information or instruction due to perceived burden and burnout. Our study found that while some contributors to vaccine fatigue are rooted in limitations of vaccine sciences and therefore can hardly be avoided, effective and empathetic vaccine communications hold great promise in eliminating preventable vaccine fatigue across sectors in society.

## Introduction

Omicron scares and speculations are gaining momentum (1, 2). While much remains unknown about the new variant of concern (3), especially in light of the unknowns associated with the BA.2 lineage of the Omicron variant (4), as evidence continues to accumulate, it is becoming clearer that mass vaccination might be one of the best defense mechanisms society has against the outbreaks (5). However, vaccine fatigue may compromise people’s vaccination intention. It is important to note that the nonstop emphasis on the importance and imperative of COVID-19 vaccines has lasted as long as the pandemic has catapulted into a public health crisis (6). In an analysis of 7,000 publishers of content in English in 2021, researchers found that among the 275 million hours people spent on reading about the most discussed topics, stories about vaccines accounted for 43 million of the total hours, whereas an additional 27 million reading hours were spent on content related to various variants of the SARS-CoV-2 virus (7).

The emergence of Omicron in the Northern Hemisphere’s winter season further suggests that the diverse and dividing debates and discussions about COVID-19 vaccines could become even more intense, if not polarizing (8). Ranging from issues centering on vaccine efficacy, vaccine equity, to the need for booster shots, the accumulated burden and burnout that are resulted from various calls for action could further deepen people’s “vaccine fatigue” (9–11). To make situations worse, confusing and conflicting media reports about vaccination may further complicate the situation. Across the pandemic continuum, chaotic reporting or corrosive informatics on COVID-19 vaccines seen in a wide array of media platforms could often be described as unreliable, unfounded, distorted, to deadly 12–14). Take the United States Centers for Diseases Control and Prevention for instance. As of February 3, 2022, a time when booster shots are part of the vaccination regime, the agency still keeping people confused about what means to be “fully vaccinated” (14). The agency’s most updated (as of January 16, 2022) definition states that “fully vaccinated means a person has received their primary series of COVID-19 vaccines” (15), which is in direct contrast with the agency’s recommendations given by government and health officials like Dr. Anthony Fauci (16). This confusion, along with other competing directives and reports (17, 18), in turn, could increase the public’s skepticism about vaccination, and in turn, add avoidable stress to personal and public health (19), let alone their harms on pandemic control and prevention.

It is important to note that, though it has been poorly investigated, vaccine fatigue is not a new phenomenon (20). Vaccine fatigue could be particularly pronounced amid large infectious disease outbreaks, largely due to people’s pronounced needs to balance the burden and burnout associated with vaccination and “social conscience, solidarity, and feelings of duty” (21). However, due to a lack of research, little is known about what factors shape people’s vaccine fatigue. The importance of understanding the antecedents to vaccine fatigue is twofold. First, an in-depth understanding of the factors that cause or catalyze people’s vaccine fatigue can help government and health officials better address the issue. Overall, without knowing what factors influence people’s vaccine fatigue, it can be extremely difficult for stakeholders to develop evidence-based interventions to reduce vaccine fatigue’s impacts on mass vaccination. Furthermore, not having a comprehensive understanding of the phenomenon and developing countermeasures against it could cause vaccine fatigue to progress into more permanent forms of vaccination non-adoption, such as vaccine hesitancy or hostility (22). This, in turn, could further hinder society’s mass vaccination efforts. Thus, to bridge the research gaps, this study aims to examine the antecedents that cause or catalyze people’s vaccine fatigue, and highlight potential solutions that could help alleviate vaccine fatigue in society.

## Methods

**Figure 1:**
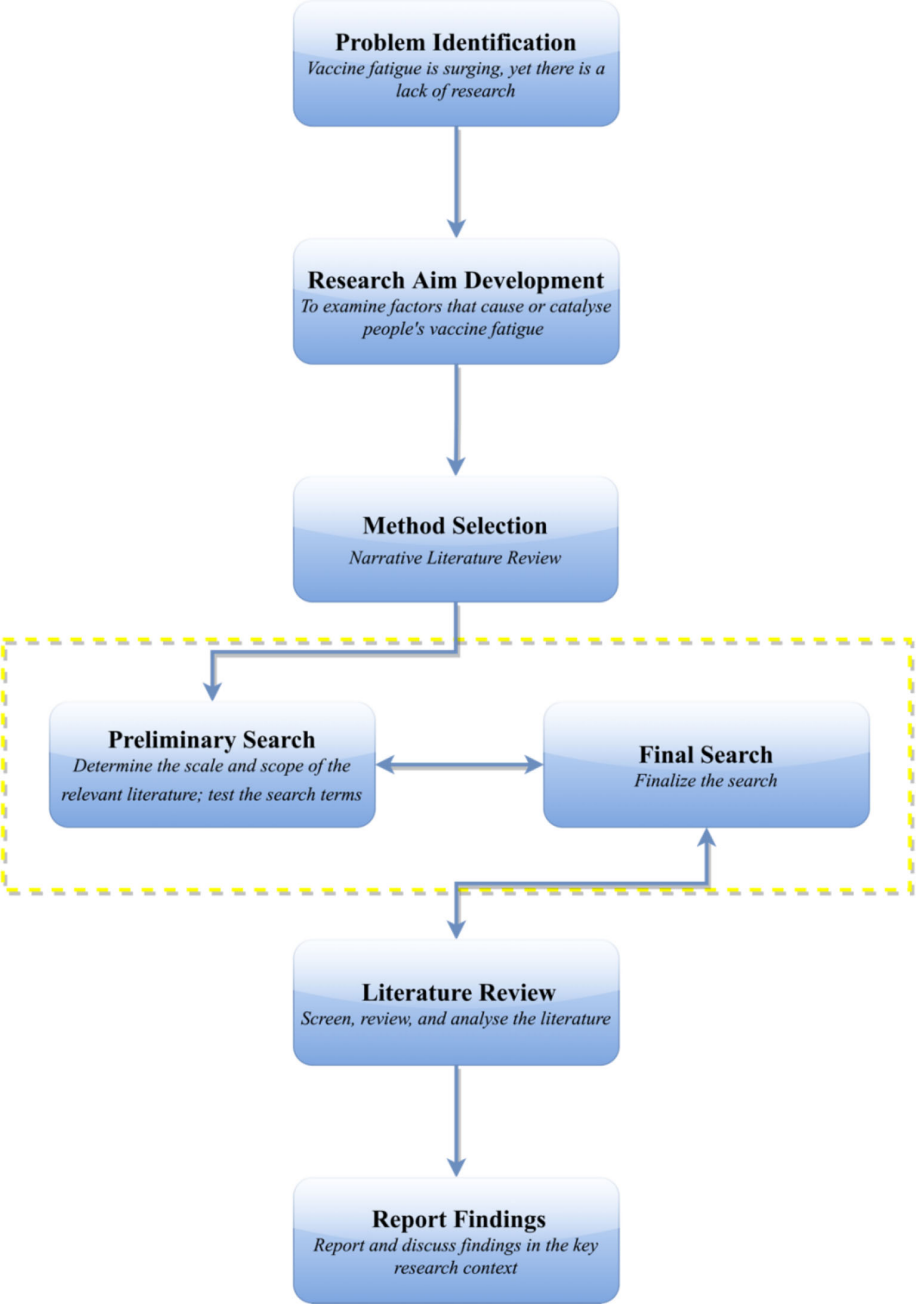
The research process in a flowchart.

A literature review was conducted in PubMed, Scopus, and PsycINFO to identify factors that introduce or intensify vaccine fatigue in society. The preliminary search was conducted on December 2, 2021, with the final search completed on December 6, 2021. All scholarly papers that addressed “vaccine fatigue”, “vaccination fatigue”, or “immunization fatigue” were examined. Records were excluded if: (1) they were not published in English, (2) the vaccines studied were not for humans [e.g., for dogs (23, 24) and poultry (25, 26)], and (3) they did not provide full text for review. In terms of theoretical framework, the narrative literature review approach was adopted, which could be understood as “an objective, thorough summary and critical analysis of the relevant available research and non-research literature on the topic being studied” (27). One key advantage of the narrative literature review approach is that it can help the researchers gain a systematic and structural understanding of what could be scattered literature in an effective manner (27). A flowchart of the research process could be found in **Figure 1**.

## Results

Overall, among all of the 47 articles found *via* the targeted search, ten papers were excluded for not meeting the eligibility criteria. A total of 37 articles were included in the final review and analysis, which yielded 70 combined occurrences of the phrases “vaccine fatigue”, “vaccination fatigue”, and “immunization fatigue” (20–22, 28–61).

Except for two pre-prints (at the time of the review) (31, 57), all of the included papers are peer-reviewed. The terms were most frequently discussed in the context of infectious diseases in general (N=11; 29.7%), followed by measles, mumps, and rubella (N=6; 16.2%), COVID-19 (N=6; 16.2%), and polio (N=5; 13.5%). The phenomenon was mainly discussed in the context of pre-vaccination, with only five articles focusing on post-vaccine fatigue syndrome (N=5; 13.5%). Most of the studies utilized the term to refer to vaccine fatigue among the public, with only two articles discussed the phenomenon from the doctors’ perspectives (45, 51), and one included both parents and doctors’ points of view (38). As detailed in **Table 1**, a large number of articles only briefly mentioned the phrases, with limited to no insights into their antecedents offered (N=16; 43.2%).

**Table 1:** A summary of key insights from the reviewed articles.

Author	Year	Disease	Title	Antecedent
Balinska et al. (30)	2004	Infectious diseases in general	Vaccine fatigue (Occurrence: 2)	NA
Humiston et al. (38)	2009	Infectious diseases in general	Vaccine fatigue (Occurrence: 10)	Increased numbers of vaccination requests (Parents) The non-fatal nature of the diseases Frequent recommendation changes and challenges in a short time (parents and doctors)
Jones et al. (39)	2007	Infectious diseases in general	Post-immunization fatigue (Occurrence: 8)	Adverse effects of vaccination (post-vaccination fatigue)
Mäding et al. (43)	2015	Infectious diseases in general	Vaccine fatigue (Occurrence: 1)	NA
Masson et al. (44)	2017	Infectious diseases in general	Vaccine fatigue (Occurrence: 1)	Adverse effects of vaccination (post-vaccination fatigue)
Scherließ (48)	2011	Infectious diseases in general	Vaccination fatigue (Occurrence: 1)	NA
Schulze et al. (49)	2016	Infectious diseases in general	Vaccination fatigue (Occurrence: 1)	Adverse effects Decreased perceived threats of the infectious diseases
Smith(20)	1997	Infectious diseases in general	Vaccination fatigue (Occurrence: 1)	“Public apathy”
Soltani (51)	2007	Infectious diseases in general	Vaccination fatigue (Occurrence: 1)	Fatigue in the doctors to motivate people to vaccinate
Thießen (55)	2016	Infectious diseases in general	Vaccination fatigue (Occurrence: 1)	NA
Thießen (21)	2021	Infectious diseases in general	Vaccination fatigue (Occurrence: 6)	Fear of side effects Disinterest Negligence
Abbas et al. (28)	2021	COVID-19	Vaccination fatigue (Occurrence: 1)	Adverse effects of vaccination (post-vaccination fatigue)
Bhopal et al. (31)	2020	COVID-19	Vaccination fatigue (Occurrence: 1)	Adverse effects of vaccination (post-vaccination fatigue)
Blanchard-Rohner et al. (32)	2021	COVID-19	Vaccination fatigue (Occurrence: 1)	NA
Iserson (22)	2021	COVID-19	Vaccine fatigue (Occurrence: 1)	Poor efficacy Being tired of constant empty promises
Rahman et al. (46)	2021	COVID-19	Vaccination fatigue (Occurrence: 1)	NA
Wagner et al. (57)	2021	COVID-19	Vaccine fatigue (Occurrence: 1) Vaccination fatigue (occurrence: 1)	NA
Bögeholz et al. (33)	2020	Measles, mumps, and rubella	Vaccine fatigue (Occurrence: 1)	NA
Coombes (34)	2009	Measles, mumps, and rubella	Vaccine fatigue (Occurrence: 1)	Too many demands of immunization
Hayashi (37)	2016	Measles	Vaccine fatigue (Occurrence: 1)	NA
Kucher (44)	2019	Measles	Vaccine fatigue (Occurrence: 3)	Perceived incidence and the likelihood of secondary diseases Persisting misinformation and beliefs Lack of relevance due to medical, religious, and/or philosophical exemptions
Stadlmann et al (52)	2011	Measles	Vaccine fatigue (Occurrence: 1)	NA
Storr et al. (54)	2018	Measles	Vaccination fatigue (Occurrence: 1)	NA
Furey (35)	2017	Polio	Vaccination fatigue (Occurrence: 1)	Misunderstandings about the vaccine’s safety and effectiveness
Mayr (45)	2006	Polio	Vaccination fatigue (Occurrence: 1)	Physical burnout of the doctors
Scott et al. (50)	2021	Polio	Vaccination fatigue (Occurrence: 1)	Risk ranking Repeated vaccination efforts
Toole (56)	2016	Polio	Vaccination fatigue (Occurrence: 2)	Too many vaccinations
Yadav et al (60)	2009	Polio	Immunization fatigue (Occurrence: 1)	Adoption of seemingly similar vaccines
Bağcı et al. (29)	2016	Ticks, borrelia burgdorferi and lyme disease	Vaccination fatigue (Occurrence: 1)	Adverse effects of vaccination (post-vaccination fatigue)
Kunze et al. (42)	2015	Tick-borne encephalitis and influenza	Vaccination fatigue (Occurrence: 1)	NA
Kunze (41)	2011	Tick-borne encephalitis	Vaccination fatigue (Occurrence: 6)	Misconceptions about the disease
Haggard (36)	2008	Pneumococcal	Vaccine fatigue (Occurrence: 1)	The non-fatal nature of the disease
Welte (58)	2016	Pneumonia-related diseases	Immunization fatigue (Occurrence: 2)	Poor understanding of the issue Lack of quality public education Opposition to the immunization principle
Zakikhany et al. (61)	2012	Diphtheria	Vaccine fatigue (Occurrence: 1)	NA
Reinheimer et al. (47)	2012	H1N1v	Vaccination fatigue (Occurrence: 1)	NA
Woodward (59)	2012	Influenza pandemic	Vaccination fatigue (Occurrence: 1)	Low mortality rates associated with the pandemic
Stafne (53)	2014	Swine flu	Vaccine fatigue (Occurrence: 1)	Lack of trust in the government’s advice about vaccination Perceive mass vaccination as exaggerated
Körber et al. (40)	2008	Tetanus	Vaccination fatigue (Occurrence: 1)	NA

NA, None.

## Discussion

This study set out to examine key factors that cause or catalyze people’s vaccine fatigue. This is the first research that systematically investigated the concept and phenomenon of vaccine fatigue. Vaccine fatigue could be understood as people’s inertia or inaction towards vaccine information or instruction due to perceived burden and burnout. By providing an in-depth and comprehensive understanding of the factors that form or fuel vaccine fatigue in society, the insights of the study can help government and health officials better design and develop countermeasures to limit the presence and prevalence of vaccine fatigue. Furthermore, our study could also help society prevent vaccine fatigue from progressing into worse forms of vaccine non-adoption (e.g., vaccine hostility), and in turn, contribute to the acceleration of mass vaccination in light of the Omicron scares and beyond. Overall, a wide range of antecedents to vaccine fatigue has been identified, ranging from the frequency of immunization demands, vaccine side effects, misconceptions about the severity of the diseases and the need for vaccination, to lack of trust in the government and the media.

### Better Vaccine Science Is Needed

Largely due to the prevalence of infectious diseases and limitations to current medical sciences, vaccine fatigue may be difficult to avoid in certain circumstances. Our findings show that many antecedents of vaccine fatigue are rooted in flaws in vaccine sciences, such as adverse effects caused by vaccination (28, 29, 31, 39, 44), poor vaccine efficacy (21, 22, 49), as well as too many vaccination demands in a relatively short period of time (34, 50, 56, 60). A study on people who received the BioNTech-Pfizer doses, for instance, found that 83.3% of the vaccine recipients experienced post-vaccination fatigue (28). In countries such as Pakistan, many children may have already received 15 doses of vaccines, which could result in vaccine fatigue in both the children and the parents (56). The limitations in current vaccine technologies are also reflected in the similarity of the vaccines people are requested to adopt, which could further fuel vaccine fatigue in the public (60).

The effects of this “too much can be an overdose” phenomenon are also felt by healthcare professionals like doctors, in which case they could become too exposed to the ever-present imperative to motivate people to vaccinate (51), or experience too much physical burnout to carry out the tasks (45). Our findings bear great implications for the current pandemic, particularly in light of the knowns and unknowns about the compounding effects of the Omicron variant and the influenza virus (62). Even before the identification of Omicron, many societies across the world have already demanded the public to take a 3-dose regime for additional protection, often with conflicting and confusing guidelines and recommendations (63), within a timeframe and a digital reality where the promise of “one dose is enough” is still making echoes (64, 65). In other words, it is possible that, with increased urgency for mass vaccination that is fueled by concerns about Omicron and the influenza-induced syndemics (66), people’s vaccine fatigue may become more pronounced and prevalent.

These insights combined, overall, highlight the need for greater investments in vaccine sciences and technologies, so that more user-friendly vaccines, in terms of the overall efficacy, duration of their protection, and the logistics associated with dose administration, could become available to the public. From the dose administration perspective, for instance, vaccines that are less invasive, such as inhalable vaccines, edible vaccines, and skin-based immunization (67–69), may also hold promise in easing people’s vaccine fatigue. It is important to note that, as our findings suggest, the public’s perceived burden and burnout that cause or catalyze their vaccine fatigue could both be psychological and physical. Therefore, in addition to developing more competent vaccines, the government and health officials should also be more mindful about how vaccine communications are designed, developed, and deployed.

### Ineffective Vaccine Communications

Our findings indicate that vaccine communications may play a critical role in shaping people’s vaccine fatigue. Overall, a wide range of antecedents to people’s vaccine fatigue is rooted in the lack of effective vaccine communications, ranging from misconceptions about vaccine efficacy and effectiveness (35, 58), poor understanding about the severity of the diseases or the urgency for vaccination (36, 41, 49, 50, 59), and erroneous or contradictory beliefs that hinder vaccination (44, 53). One way to address this issue, as indicated by previous research, is *via* developing effective education and communication programs, such as persuasive advertising campaigns (20, 21), as opposed to compulsory interventions. A key consideration for not employing stringent, if not draconian, measures to address people’s vaccine fatigue centres on the possibility that they may result in unintended consequences such as stigmatization or discrimination in the public (21, 55).

This consideration is in line with our findings, which suggest that rather than a permanent trait found in populations such as anti-vaxxers, vaccine fatigue broadly represents a transitory stage that is more common in people who hold a pro-vaccination view. Considering that compulsory interventions may result in potential consequences such as forcing the momentary vaccine fatigue into more aggressive and permanent forms of vaccine non-adoption, such as vaccine hostility, it is important that government and health officials adopt an empathetic approach to vaccine communications (70). This is particularly important considering that many nations worldwide have implemented or are considering establishing vaccine mandates amid COVID-19, even though considerable public backlash is present (71–73). The need for mindful and compassionate interactions with the public might be particularly pronounced in light of the potential unintended consequences vaccine communication could cause.

### Lack of Trust

This study’s findings show that a lack of trust in the government and the media is also an antecedent to vaccine fatigue (53). As a vital bridge between science, scientists, and the public throughout health emergencies like COVID-19, the media industry is a critical link in society’s collective defence against the pandemic. Take COVID-19 for instance. Across the pandemic, both legacy media and social media platforms have played an indispensable role in informing almost every aspect about COVID-19 and more (74). However, what is also evident in the pandemic is that, due to the prevalence of COVID-19 infodemics and the polarizing role many media outlets have endorsed (12, 19, 75). The public’s trust over media reports on COVID-19, such as COVID-19 vaccines, has been deteriorating (76, 77).

It is also important to note that, even without the highly mediated influence of the news reports and analyses, the sheer scale and scope of negative events induced by COVID-19 may be enough to cause discomfort and distress in the public (19). It is possible that, to avoid potential or additional stress caused by the media reports on COVID-19 vaccines, people might develop a passive attitude towards news about the vaccines and the shots themselves, in the form of vaccine communication avoidance, and in turn, vaccine fatigue. In light of the dearth of research and the outsized influence of media on public’s health behaviors amid the beyond COVID-19 (78), more research and interventions are needed to ensure a healthy and symbiotic relationship can be formed between government and health officials, media professionals, citizen journalists, and the public in the context of vaccine communications.

### Limitations

While our study bridges important gaps in the literature, it is not without limitations. For starters, we only reviewed and analyzed scholarly literature, which means that insights such as relevant media reports were not included in the review. Furthermore, only academic literature in English was considered in the current study. In light of the multifaceted nature of vaccine fatigue and its implications, future research could consider extending current understandings on vaccine fatigue by conducting country- or language-specific investigations on the phenomenon.

## Conclusion

Vaccine fatigue is detrimental to both personal and public health. Our study found that effective vaccine communications hold great promise in limiting vaccine fatigue among key stakeholders. The role of trust is also incremental to shaping people’s compliance with vaccine-related directives, which in turn, further emphasizes the importance of developing safer and more efficacious vaccines, along with responsible and accountable public health directives in mitigating vaccine fatigue. Overall, in light of the knowns and unknowns about COVID-19, ranging from the high transmissibility of Omicron to the lack of knowledge about Omicron’s variant—BA.2, in order to effectively prevent vaccine fatigue from plaguing pandemic control and prevention efforts, more endeavors are needed to understand the causes and consequences of vaccine fatigue amid COVID-19 and beyond.

## Author Contributions

ZS conceived the work, reviewed the literature, drafted, and edited the manuscript. AC, DM, CV, and Y-TX reviewed the literature and edited the manuscript. All authors contributed to the article and approved the submitted version.

## Funding

This work was supported by FHS Faculty funding.

## Conflict of Interest

The authors declare that the research was conducted in the absence of any commercial or financial relationships that could be construed as a potential conflict of interest.

## Publisher’s Note

All claims expressed in this article are solely those of the authors and do not necessarily represent those of their affiliated organizations, or those of the publisher, the editors and the reviewers. Any product that may be evaluated in this article, or claim that may be made by its manufacturer, is not guaranteed or endorsed by the publisher.
