# Corrigendum: Mind the “vaccine fatigue”

**DOI:** 10.3389/fimmu.2022.1122354

**Published:** 2023-01-04

**Authors:** Zhaohui Su, Ali Cheshmehzangi, Dean McDonnell, Claudimar Pereira da Veiga, Yu-Tao Xiang

**Affiliations:** ^1^ School of Public Health, Institute for Human Rights, Southeast University, Nanjing, China; ^2^ Department of Architecture and Built Environment, Architecture and Urban Design, Faculty of Science and Engineering, University of Nottingham Ningbo China, Ningbo, China; ^3^ Network for Education and Research on Peace and Sustainability, Hiroshima University, Hiroshima, Japan; ^4^ Department of Humanities, South East Technological University, Carlow, Ireland; ^5^ School of Management—PPGOLD, Federal University of Parana—UFPR, Curitiba, Brazil; ^6^ Unit of Psychiatry, Department of Public Health and Medicinal Administration, University of Macau, Macao, Macao SAR, China; ^7^ Institute of Translational Medicine, Faculty of Health Sciences, University of Macau, Macao, Macao SAR, China; ^8^ Centre for Cognitive and Brain Sciences, University of Macau, Macao, Macao SAR, China; ^9^ Institute of Advanced Studies in Humanities and Social Sciences, University of Macau, Macao, Macao SAR, China

**Keywords:** COVID-19, vaccination, vaccine fatigue, public health, vaccine communications

In the published article, there was an error in affiliation 1. Instead of “Center on Smart and Connected Health Technologies, Mays Cancer Center, School of Nursing, UT Health San Antonio, San Antonio, TX, United States”, it should be “School of Public Health, Institute for Human Rights, Southeast University, Nanjing, China.”

In the published article, there was an error in affiliation 4. Instead of “Department of Humanities, Institute of Technology Carlow, Carlow, Ireland”, it should be “Department of Humanities, South East Technological University, Carlow, Ireland.”

In the published article, there was an error in affiliation 6. Instead of “Unit of Psychiatry, Department of Public Health and Medicinal Administration, Faculty of Health Sciences, University of Macau, Macao, Macao SAR, China”, it should be “Unit of Psychiatry, Department of Public Health and Medicinal Administration, University of Macau, Macao, Macao SAR, China.”

The authors apologize for these errors and state that they do not change the scientific conclusions of the article in any way. The original article has been updated.

